# Malignant hyperthermia-like syndrome in acute chlorfenapyr poisoning – A case report

**DOI:** 10.1016/j.heliyon.2022.e10051

**Published:** 2022-08-04

**Authors:** Shuyi Zhang, Yuxiao Deng, Yuan Gao

**Affiliations:** Department of Intensive Care Medicine, Renji Hospital Affiliated to Shanghai Jiao Tong University School of Medicine, Shanghai, China

**Keywords:** Chlorfenapyr, Insecticide, Poisoning, Malignant hyperthermia, Mitochondrial disorders

## Abstract

**Background:**

Chlorfenapyr is a pesticide that interferes with mitochondrial oxidative phosphorylation, resulting in the disruption of ATP production and cellular death. We present a fatal case of chlorfenapyr poisoning presented with malignant hyperthermia- like syndrome after intubation.

**Case presentation:**

A 49-year-old male presented with fatigue and diaphoresis four days after ingesting emamectin chlorfenapyr. IV hydration was given, and two sessions of hemoperfusion were performed. He was intubated for airway protection on Day 3 because of drowsiness. Immediately after intubation, he developed tachycardia and hyperthermia (temperature 41 °C), followed by cardiac arrest. During resuscitation, we noted he had severe diaphoresis and generalized muscle rigidity. Peri-arrest ABG showed abrupt onset of severe type 2 respiratory failure, lactate acidosis, and hyperkalemia. The clinical manifestation and ABG result lead to the possible diagnosis of malignant hyperthermia. The resuscitation was unsuccessful, and the patient eventually passed away. Propofol might be the culprit drug in this case as it is known to affect mitochondrial metabolism via uncoupling oxidative phosphorylation.

**Conclusion:**

We suggest monitoring for signs and symptoms of malignant hyperthermia in chlorfenapyr poisoning, especially after intubation. Propofol should be avoided or used with caution during induction for intubation. Further research on the possible antidote and usage of early RRT in ED is needed.

## 1 Introduction

Chlorfenapyr is a pesticide used to control a number of cotton pests and was approved to use more widely in vegetables and fruit ten years ago [1]. It interferes with mitochondrial oxidative phosphorylation, resulting in the disruption of ATP production and cellular death [2].

Chlorfenapyr poisoning exhibits high mortality. Fatal cases are reported in China [3, 4], India [5], Japan [6, 7], Korea [2, 8], and North America [9]. Of 15 case reports available so far, only 3 cases reported survival after intentional ingestion.

Clinical manifestation might be minimal at the early stage, especially in those with low exposure doses. However, delayed toxicity was presented six to seven days after exposure. Symptoms of toxicity include diaphoresis, hyperthermia, rhabdomyolysis, renal failure, metabolic acidosis, and decreased level of consciousness due to progressive and late-onset central nerve damage.

We present the fatal case of a 49-year-old male who ingested the chlorfenapyr in a suicide attempt and developed malignant hyperthermia- like syndrome right after intubation. This is the first case report of malignant hyperthermia in acute chlorfenapyr poisoning.

## 2 Case presentation

A 49-year-old male farmer presented to ED with fatigue and diaphoresis four days after ingesting about 30 ml of 12% emamectin chlorfenapyr for a suicide attempt. He denied ingestion of other toxic substances, medications, or alcohol. His vitals on arrival at ED were: temperature 36.6 °C, pulse 77 bpm, respiratory rate 18/min, BP 126/79 mmHg, SpO2 100% on room air. He was lucid, and his physical examinations were normal except for mild tachypnea. No neurological deficits were found. The initial lab result showed Creatine Kinase (CK) 3826 U/L(55–170 U/L), aspartate aminotransferase (AST) 161 U/L(17–59 U/L), potassium (K^+^) 3.27 mmol/L (3.5–5.1 mmol/L), NT-proBNP 334 pg/ml (<125 pg/mL). Other labs were unremarkable. ECG and Troponin I (TnI) were normal. He was given conservative management with IV fluids in ED and transferred to ICU for close monitoring due to concern of delayed chlorfenapyr toxicity.

Hemoperfusion was immediately performed to remove any residual chlorfenapyr and potential active metabolites. The patient tolerated two sessions well on Day 1 and Day 2 without any hemodynamic compromise. Raising and up-trending CK (from 3826 U/L to 5385 U/L on D3) raised the concern of rhabdomyolysis. Intravenous + oral fluids (6.5–7.5 L/day) and sodium bicarbonate was given for hydration and alkalization. The patient became increasingly drowsy and had a fever of 38.1 °C on Day 2. His GCS dropped to E02M4 on D3 and started Kussmaul breathing. We decided to intubate him for airway protection. The arterial blood gas (ABG) done 5 min before intubation was unremarkable except for type 1 respiratory failure: pH 7.442, pCO2 38.1 mmHg, pO2 60.1 mmHg, sodium (Na^+^) 145.2 mmol/L, K^+^ 3.42 mmol/L, Calcium (Ca^2+^)0.997 mmol/L, Lactate 1.7 mmol/L, bicarbonate(HCO3) 25.4 mmol/l, base excess(BE) 1.35 mmol/l.

**Figure 1:**
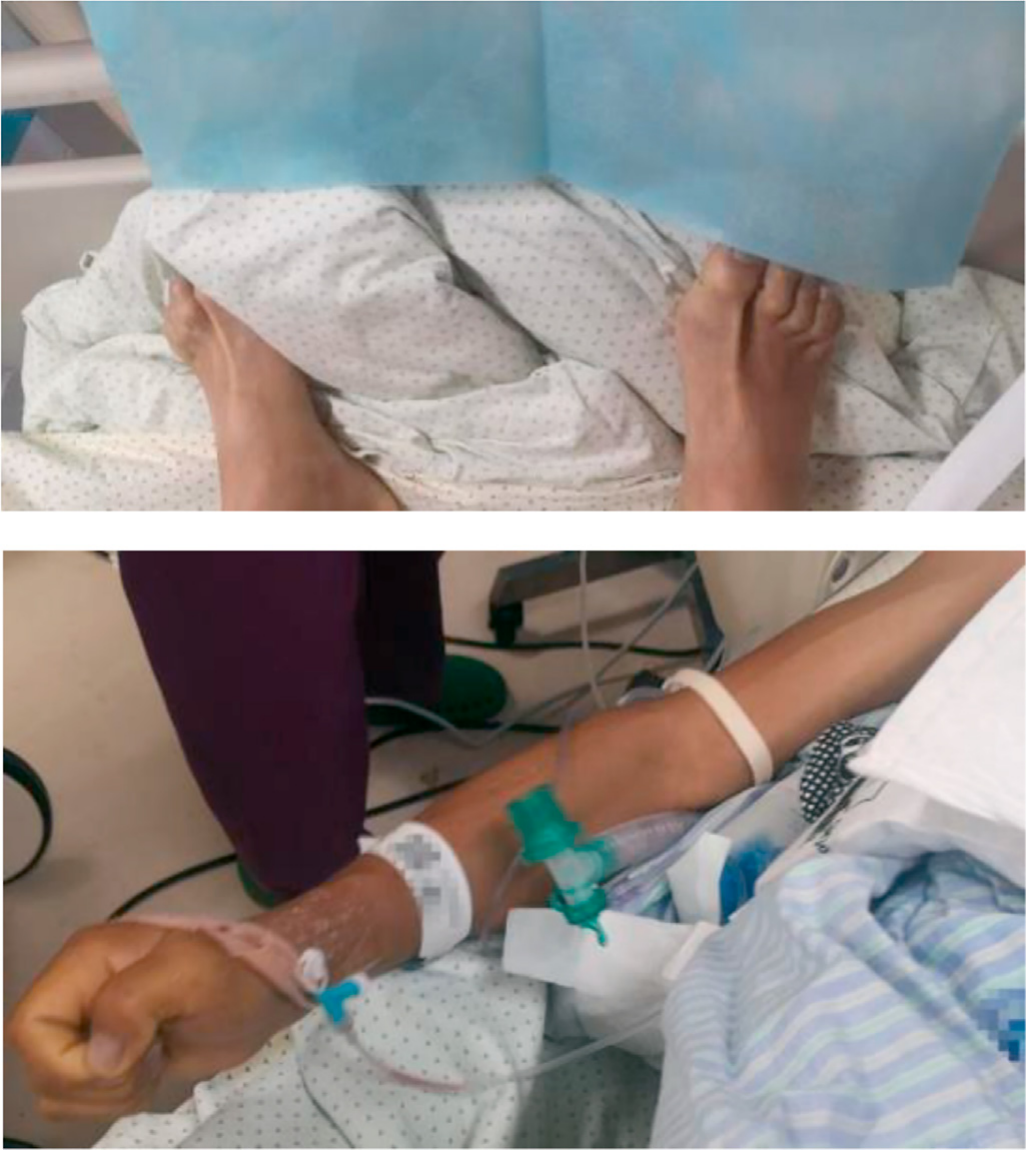
Generalized muscle rigidity during resuscitation.

IV 1% propofol 10 ml, fentanyl 100 mcg iv, and rocuronium 30 mg were given for sedation and paralysis. The intubation was successful. However, he had sinus tachycardia and hypertension during intubation. Nevertheless, only one minute after intubation, his blood pressure started to crash rapidly despite iv boluses of noradrenaline, followed by bradycardia. Within another minute, he went into PEA arrest, and ACLS was started. A repeated ABG was done to search for the cause of rapid deterioration, which showed severe type 2 respiratory failure, lactate acidosis, and hyperkalemia, PH 7.12, pCO2 66.7 mmHg, pO2 74.9 mmHg, K+ 6.91 mmol/L, Na+ 156 mmol/L, Ca2^+^ 0.881 mmol/L, Glucose 9.4 mmol/L, Lactate 12.7 mmol/L, BE -10.3 mmol/l. The remeasured temperature was 41 °C. He had severe diaphoresis and generalized muscle rigidity (Figure 1). We think the cause of the arrest was severe acidosis and hyperkalemia caused by possible malignant hyperthermia, and the initial tachycardia and hypertension was an early sign of that. Intravenous cool saline and ice pack were applied. The resuscitation was unsuccessful, and the patient eventually passed away.

Informed Consent was obtained from the patient's close relatives because the patient expired prior to the conception of this manuscript.

## 3 Discussion

Chlorfenapyr is a pro-insecticide and requires metabolic conversion to the active uncoupler, Pyrrole (also named AC 303,268, CL 303268) before it exerts a toxic action. AC 303,268 is a lipophilic weak acid with very strong uncoupling activity that uncouples oxidative phosphorylation at the mitochondria, resulting in disruption of the production of ATP, cellular death, and ultimately organism death [10, 11] The effects of the uncoupling of oxidative phosphorylation are presented as clinical symptoms, more apparent in cells with high energy demands such as cardiac muscle, skeletal muscle, retina, and the central nervous system (CNS) [12]. These mechanisms could explain the cardinal features of chlorfenapyr poisoning, including rhabdomyolysis, respiratory failure, decreased level of consciousness and intracranial and spinal cord demyelination [5].

Literature review shows that delayed hyperthermia is quite common and rapid deterioration after intubation was documented in several case reports as well as in our patient (Table 1). Once the patient went into PEA arrest, it was quite refractory. There was no clear documentation for the cause of cardiac arrest, and none of the cases reported ROSC during resuscitation. In one case the pre-arrest investigations were unremarkable except for raised CK and myoglobulin [2]. This is the first case documented that had a relatively clear cause for the cardiac arrest in chlorfenapyr poisoning.

**Table 1:** Short Time from intubation to cardiac arrest in existing case reports.

	Reason for intubation	Time from intubation to cardiac arrest	Temperature before intubation (°C)	Temperature after intubation (°C)
Case 1 [8]	Airway protection	40 min	38.5	41.5
Case 2 [2]	Decreased consciousness, for airway protection	Soon (unspecified)	-	40.9
Case 3 [3]	Desaturation, decreased consciousness	3 min	40	-
Case 4 [3]	Kussmaul breathing, desaturation	3 min	41	-
Case 5 [3]	Desaturation, decreased consciousness	Soon (unspecified)	40	-
Case 6 [3]	Desaturation, decreased consciousness	8 h	40.1	41.8

Malignant hyperthermia (MH) is a rare disorder caused by an excessive calcium release from the sarcoplasmic reticulum in skeletal muscles [13]. It's characterized by a rapid rise in body temperature, rhabdomyolysis, and if untreated, collapse and death. MH occurs when certain anesthetics (e.g. volatile anesthetic or succinylcholine) produce a rapid uncoupling of oxidative phosphorylation in susceptible individuals with autosomal dominant disorder [14]. During an acute event, there's no confirmatory test for MH. The diagnosis is based upon clinical signs of hypercapnia, tachycardia, muscle rigidity, rhabdomyolysis, hyperthermia, arrhythmia, and laboratory abnormalities (e.g. respiratory and possibly metabolic acidosis, hyperkalemia, elevated creatine kinase, serum, and urine myoglobin) [15]. Our patient exhibits an abrupt increase in potassium, paCO 2, and lactate within just 20 min. These ABG result combined with the clinical sign of hyperthermia, diaphoresis, and rigidity leads to the possible diagnosis of malignant hyperthermia. The MH likelihood is “almost certain” according to the MH clinical grading scale (71 points): generalized muscle spasm (15 points), potassium >6 mmol/L without renal failure(3 points), PCO2>60 mmHg in controlled ventilation (15 points), inappropriate rapid increase in temperature (15 points), inappropriate sinus tachycardia (3 points), base excess below -8 mmol/L (10 points) and Arterial pH < 7.25 (10 points) [16]. The confirmatory diagnosis for MH required both clinical manifestation and more specific evidence for MH susceptibility——in-vitro contracture test (IVCT) by muscle biopsy and genetic test for variants in RYR1, CACNA1S, STAC3 genes [13]. In our patient, both were not carried out due to the unavailability of those tests in our institution.

We are uncertain whether this is merely a natural disease progression that coincides with the time of intubation or was the intubation triggered the malignant hyperthermia-like syndrome.

The induction agents propofol, fentanyl, and rocuronium we used were assumed to be safe for malignant hyperthermia by MHAUS [17]. However, case reports had documented propofol or non-depolarizing neuromuscular blocking agents like rocuronium associated with MH [18, 19] Propofol is known to affect mitochondrial metabolism via uncouple oxidative phosphorylation and inhibit complexes I, II, and IV in vitro [20, 21, 22, 23] Boluses of propofol have been used safely for pre-operation induction in patients with mitochondrial disorders, but continuous infusion was not recommended. Patients who are metabolically compromised may not be able to tolerate propofol [20]. Thus we suspect propofol might be the culprit drug for the malignant hyperthermia-like syndrome based on its uncoupler characteristic may exacerbate the toxic uncoupling activity of chlorfenapyr metabolite.

So far, there is no effective antidote for chlorfenapyr poisoning. Treatment is mainly supportive. Methylene blue increases the respiration of the mitochondria in the bumblebee. Thus it is a potential antidote against the toxic action of chlorfenapyr [24]. More animal studies and further clinical trials are warranted. Late hemoperfusion and hemodialysis had been tried in previous reports with a lack of response. We observed similar futility of hemoperfusion in our patient, likely due to the lipophilic nature of chlorfenapyr active metabolites. However, early RRT before its conversion to Pyrrole is still a future consideration to reverse the clinical course.

## 4 Conclusion

We reported a fatal case of malignant hyperthermia-like syndrome in chlorfenapyr poisoning that developed shortly after intubation. We suggest monitoring for signs and symptoms of malignant hyperthermia in chlorfenapyr poisoning, especially after intubation. Propofol should be avoided or used with caution during induction for intubation. We recommend Dantrolene be prepared in advance in case symptoms of malignant hyperthermia develop. Further research is needed on the possible antidote methylene blue and usage of early RRT in ED.

## Declarations

### Author contribution statement

All authors listed have significantly contributed to the investigation, development and writing of this article.

### Funding statement

This research did not receive any specific grant from funding agencies in the public, commercial, or not-for-profit sectors.

### Data availability statement

No data was used for the research described in the article.

### Declaration of interest's statement

The authors declare no conflict of interest.

### Additional information

No additional information is available for this paper.
